# Suubi4Her: a study protocol to examine the impact and cost associated with a combination intervention to prevent HIV risk behavior and improve mental health functioning among adolescent girls in Uganda

**DOI:** 10.1186/s12889-018-5604-5

**Published:** 2018-06-05

**Authors:** Fred M. Ssewamala, Laura Gauer Bermudez, Torsten B. Neilands, Claude A. Mellins, Mary M. McKay, Irv Garfinkel, Ozge Sensoy Bahar, Gertrude Nakigozi, Miriam Mukasa, Lindsay Stark, Christopher Damulira, Jennifer Nattabi, Apollo Kivumbi

**Affiliations:** 1 Brown School, Washington University in St. Louis, 1 Brookings Drive, St. Louis, MO 63130 USA; 2 Columbia University School of Social Work, 1255 Amsterdam Ave., New York, NY 10027 USA; 3 School of Medicine, University of California San Francisco, 550 16th Street, San Francisco, CA 94158 USA; 4 Rakai Health Sciences Program, Old Bukoba Road, 279 Kalisizo, Uganda; 5 Department of Population and Family Health, Columbia University Mailman School of Public Health, 60 Haven Ave B-4 Suite 432, New York, NY 10032 USA; 6 HIV Center for Clinical & Behavioral Studies, New York State Psychiatric Institute and Columbia University, 1051 Riverside Dr, New York, NY 10032 USA; 7 International Center for Child Health and Development Field Office, Plot 23 Circular Rd, Masaka, Uganda

**Keywords:** HIV, Adolescent girls, Assets, Economic empowerment, Family strengthening, Combination interventions

## Abstract

**Background:**

Asset-based economic empowerment interventions, which take an integrated approach to building human, social, and economic capital, have shown promise in their ability to reduce HIV risk for young people, including adolescent girls, in sub-Saharan Africa. Similarly, community and family strengthening interventions have proven beneficial in addressing mental health and behavioral challenges of adolescents transitioning to adulthood. Yet, few programs aimed at addressing sexual risk have applied combination interventions to address economic stability and mental health within the traditional framework of health education and HIV counseling/testing. This paper describes a study protocol for a 5-year, NIMH-funded, cluster randomized-controlled trial to evaluate a combination intervention aimed at reducing HIV risk among adolescent girls in Uganda. The intervention, titled *Suubi4Her,* combines savings-led economic empowerment through youth development accounts (YDA) with an innovative family strengthening component delivered via Multiple Family Groups (MFG).

**Methods:**

Suubi4Her will be evaluated via a three-arm cluster randomized-controlled trial design (YDA only, YDA + MFG, Usual Care) in 42 secondary schools in the Central region of Uganda, targeting a total of 1260 girls (ages 15–17 at enrollment). Assessments will occur at baseline, 12, 24, and 36 months. This study addresses two primary outcomes: 1) change in HIV risk behavior and 2) change in mental health functioning. Secondary exploratory outcomes include HIV and STI incidence, pregnancy, educational attainment, financial savings behavior, gender attitudes, and self-esteem. For potential scale-up, cost effectiveness analysis will be employed to compare the relative costs and outcomes associated with each study arm.

**Conclusions:**

Suubi4Her will be one of the first prospective studies to examine the impact and cost of a combination intervention integrating economic and social components to reduce known HIV risk factors and improve mental health functioning among adolescent girls, while concurrently exploring mental health as a mediator in HIV risk reduction. The findings will illuminate the pathways that connect economic needs, mental health, family support, and HIV risk. If successful, the results will promote holistic HIV prevention strategies to reduce risk among adolescent girls in Uganda and potentially the broader sub-Saharan Africa region.

**Trial registration:**

Clinical Trials NCT03307226 (Registered: 10/11/17).

## Background

Sub-Saharan Africa (SSA) remains the world’s most affected region in the HIV epidemic, home to 71% of people living with HIV worldwide [1] and girls accounting for 7 out of 10 new infections among adolescents (ages 15–19 years) [2]. This gender disparity has increased recognition that adolescent girls need more attention if the global community is to achieve an AIDS-free generation. Studies and theory suggest causal pathways between family economic resources, education, mental health, and HIV risk [3, 4]. Being out-of-school is one of the key characteristics found to increase young women’s vulnerability to HIV as it is associated with numerous risk factors, including age-disparate and transactional sex, early marriage, inconsistent condom use, and limited power in relationships – most significantly the ability to negotiate safe sex [5–11]. Alongside these risks exist mental health challenges associated with economically motivated sex (both age-disparate and transactional), which have been shown to have a bi-directional relationship with depression, low self- esteem, and anxiety for young women [12, 13]. Moreover, higher depression among young females has been associated with co-factors of HIV risk [14]. Given the heightened risk for HIV infection in adolescent girls, there is an urgent need to address the complex and multilayered economic and psychosocial issues facing this population in SSA.

In many SSA countries, including Uganda, access to education remains strongly associated with household economic stability [15]. Lack of financial resources is the most commonly cited reason why adolescent girls fail to attend school [16–19]. Moreover, cultural norms can be influential and families may feel pressure to prioritize male education when resources are few. Several traditions in SSA are passed down generationally and encourage stratification of gender roles, such as adolescent marriage and early childbearing, both of which can prompt separation from school for adolescent girls [20–22].

At the same time, family economic stability influences the quality of family relationships with poverty adversely impacting parent-child communication and involvement [23–25]. Studies have documented that strong positive connections and more open communication between a child and his/her primary caregiver can predict mental health outcomes, delays in onset of sex, and overall child adjustment [26–31]. Additionally, better parenting skills have been associated with adolescents having less susceptibility to peer pressure [31, 32]. Thus, particularly in low-resource settings, supporting families with economic opportunities and strengthening family supportive processes may minimize risk taking behaviors, discourage school separation, and address mental health stressors among adolescents.

Given the complex and multi-dimensional drivers of increased HIV risk among adolescent girls in SSA and the failure of most single interventions to significantly decrease these rates, investments in combination interventions are critical to provide an interdisciplinary, multi-level response needed to reduce new HIV and STI infections in a way that single interventions alone have not yet been able. To address the identified challenges, this trial, entitled “Suubi4Her” (also known as *Hope for Girls)*, aims to examine the impact of and cost associated with an innovative combination intervention on HIV risk behaviors among adolescent girls. The intervention is composed of two programs: 1) Asset-based financial inclusion (specifically youth development accounts – (YDAs), and 2) An evidence-based, family strengthening approach to enhancing youth behavioral health delivered via multiple family groups (MFG). Designed within a prevention framework, the Suubi4Her intervention seeks to support vulnerable adolescent girls before they drop out of school when their exposure to HIV-risk taking behaviors increases. The integration of efforts towards strengthened family relationships and improved communication among household members may be crucial in helping families navigate adolescent transitions to adulthood. Suubi4Her pairs two innovative and evidence-based interventions together, an asset-based financial inclusion/economic strengthening model and a family strengthening approach to enhancing youth behavioral health delivered via MFGs, recognizing the possibility that mental health may be a critical component intersecting between poverty and HIV risk for young females.

## Methods

### Study aims

The study aims are to: 1) Examine whether the Suubi4Her intervention is effective in protecting adolescent girls against known HIV risk factors; 2) Elucidate the effects of the Suubi4Her intervention on mental health functioning and examine the effects of these variables as potential mechanisms of change, mediating the relationship between each intervention and HIV risk reduction; and 3) Evaluate the cost-effectiveness of each intervention condition.

### Setting

Over the course of five years (August 2017 – July 2022), Suubi4Her will be implemented in the Central Region of Uganda, an area heavily affected by HIV with a prevalence rate of 10.6%, three percentage points higher than the national average of 7.4% [33]. In 2013, the number of new HIV infections for adolescents (ages 15–19) in Uganda was twice as high for girls (est. 10,000) as it was for boys (est. 4500) [34]. With data from 2014, UNAIDS reported that 570 adolescent girls and young women (ages 15–24) were acquiring HIV per week in Uganda [1].

### Study population, recruitment, and retention

A total of 1260 secondary school-going girls (ages 15–17 at enrollment) in their first year of secondary school will be enrolled and followed for four years. Given the high incidence of HIV in the study area and to avoid stigma that surrounds being HIV positive in the region, no girl will be excluded by virtue of her HIV status. Given the high prevalence of HIV in our proposed study sample, to further ensure that HIV serostatus is balanced across interventions, HIV status will be used as a blocking factor during the cluster randomization process using the strategy outlined in Bellamy et al. [35].

Adolescents will be included within the study if they meet the following criteria: 1) female; 2) enrolled in first year of secondary school in Rakai, Kyotera, Masaka, Lwengo or Kalungu districts; 3) age 15–17 years; 4) living within a family (broadly defined and not an institution or orphanage, as those in institutions have different familial needs). Exclusion criteria: Girls are ineligible if: 1) they have a severe cognitive or severe psychiatric impairment that would prevent comprehension of study procedures as assessed by trained staff during the Informed Consent process; or 2) they are unwilling or unable to commit to completing the study. We will not exclude girls because of their HIV, STI and/or pregnancy status. Analysis will be adjusted to account for these baseline factors.

Using the same recruitment procedures tested in three earlier studies (Suubi+Adherence R01HD074949, Bridges to the Future R01HD070727 and Suubi Maka R34MH081763), schools and the local district administration will be relied upon to identify participants and help with recruitment. Existing school enrollment procedures will be leveraged to invite caregivers with an eligible child to contact the school for further details. In addition, community development officers and implementing partners will distribute flyers during their frequent community visits to inform caregivers whose children meet the inclusion criteria but may not yet have reported back to school. Adolescents and caregivers who indicate interest will be invited to come to the school in-person for a one-on-one information meeting with the research team, during which they will be given details of the project.

The project will take place in a highly stable region of Uganda where geographical moves are rare, facilitating ability to track and retain the sample. We will ask participants to give the telephone number, names, addresses, and contact information for three people who will always know how to reach them. Participants will be told that if we contact the people listed, we will not discuss any details about them or their study participation. We will use these records to help track their location only if we have lost contact. We will also be in contact with all participants regularly during school roll calls to determine enrollment and attendance. Moreover, we will have monthly contact with children in the two treatment conditions through the distribution of their monthly savings banks statements. This frequent contact will enable the research team to continually engage all participants, and minimizing loss to follow-up. Based on prior studies in the same region, we expect attrition by end of follow-up to be no more than 17%.

### Intervention description

**Fig. 1:**
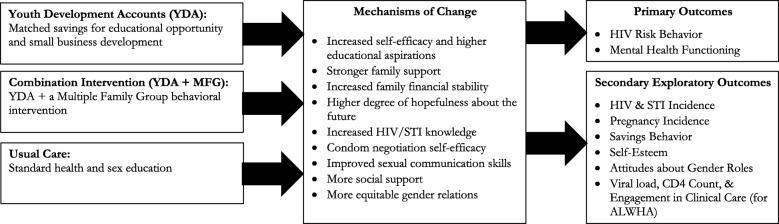
Suubi4Her Study Design

The study has been designed as a three-arm, cluster randomized-controlled trial (RCT), consisting of two treatment arms and one control arm (Fig. 1).

### Treatment arm 1 - YDA

YDAs are guided by asset-theory [3, 4] which posits that financial and tangible assets not only impact economic stability for individuals and households but also have important developmental and psychological benefits including future-oriented thinking, feelings of security, and self-efficacy. The argument advanced by asset-theory is consistent with several behavioral and psychosocial theories that have guided studies on sexual risk-taking and mental health, including Bandura’s Social Cognitive Theory [36] and the Theory of Reasoned Action [37].

Adolescents in both treatment arms will be enrolled in a 1:1 matched savings program at a national financial institution. Each account will be in the name of the adolescent, with her primary caregiver as a co-signer, until she turns 18 years of age, at which time a co-signer will no longer be required. This is consistent with the Ugandan banking law which prohibits children below age 18 from independently entering into a binding contract and operating a bank account. The matching funds will be kept in a separate account from the participants’ own savings. When a girl is ready to pay for school fees, the check for the matching funds will be written in the name of the school she attends or directly wired to the school’s bank account. This process is intended to eliminate the risk of family pressure on the girl to withdraw money set aside for education and skills training.

Participating students will be allowed to use up to 30% of their total matched savings to invest in a family-based income-generating activity (IGA). The remaining 70% of the savings will be restricted to fund the education and skills training of participating adolescent girls. Consistent with earlier studies, a participant’s access to the matching funds is conditional upon completion of 12 financial management workshops over 12 months. The workshops, to be implemented by collaborating community agency, Reach the Youth-Uganda, in collaboration with the financial institutions holding the YDAs, will consist of 12 general workshops that cover basic principles of financial management including income generation, use of financial institutions, saving, and asset-building.

### Treatment arm 2 – Combination intervention (YDA + MFG)

The combined treatment arm will consist of a YDA (detailed above) and a family-based dialogue and training delivered via MFGs that aim to strengthen family relationships and address mental health challenges that commonly occur in adolescence.

The MFG has adopted the strengths of multiple therapeutic methods and theories to create an extremely flexible approach which has been applied to a variety of target populations struggling with a diverse range of issues [38–40]. MFG is based on building support for families by providing opportunities for parents and children to communicate in a safe setting with other families who have shared experiences thus allowing each family to benefit from the contributions of one another [40]. Advice and insight from other families is often seen as less threatening than feedback given by a therapist [40]. In addition, MFG focuses on reducing stigma by normalizing shared experiences. The MFG intervention acknowledges poverty as a stressor that may undermine parenting while also recognizing the contextual challenges that contribute to poor mental health functioning for adolescent girls, including high rates of poverty, violence, and family loss due to HIV and other health threats [41–43]. The MFG approach will allow girls and their families to share their experiences with others in similar situations, thus building hope by providing social support, normalization of similar experiences and struggles, and the sharing of effective solutions [44]. Suubi4Her will utilize the MFG approach to specifically target family communication, support, and gender equality both within and across families from the same community setting.

### Control arm – Usual care

In Uganda, an Adolescent Sexual and Reproductive Health curriculum is required of all secondary school students [45]. As such, these curricula will be considered usual care and all enrolled participants, both in control and treatment conditions, will be exposed. The Adolescent Sexual Reproductive Health content is dispersed across a range of academic subjects in secondary schools. In each class, students receive information about sexual activity, HIV prevention, and gender studies relevant to that subject. Teachers and students all receive a sex and health education handbook. The content related to HIV and sexual risk-taking behaviors includes delaying sex; using condoms and contraception; preventing forced sex; and preventing substance abuse. This curriculum also includes education on gender equality and importance of delayed marriage. Prior to study implementation, the research team will hold induction meetings for all teachers involved in the study to ensure uniform delivery of the Ministry of Education approved sex education curriculum.

### Randomization, sample size, and power analysis

**Fig. 2:**
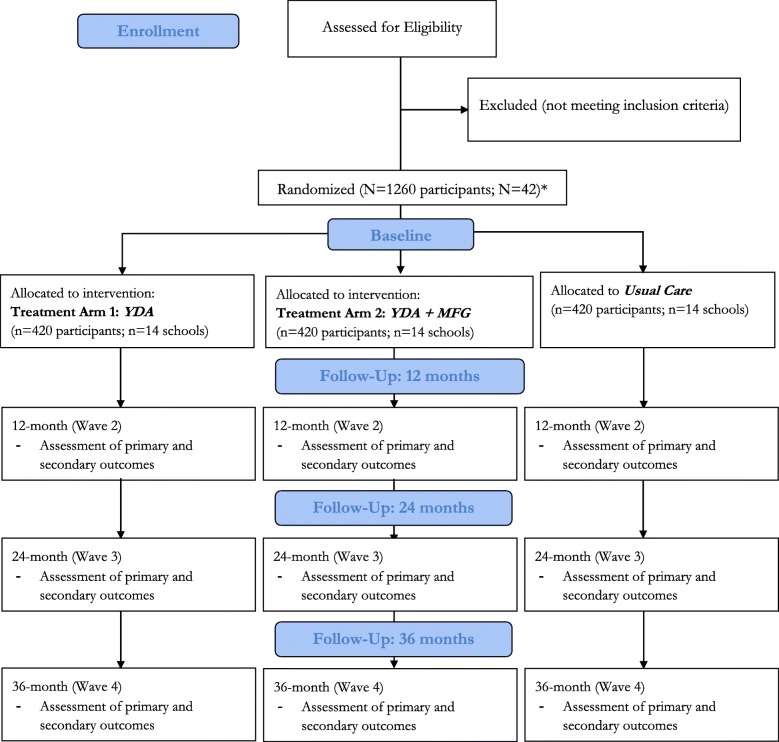
CONSORT Flow Diagram – Suubi4Her

Stratified random sampling will be used to allocate schools to four strata based on two variables: 1) student population size (medium size vs. large), and 2) geographical location (rural vs. urban), to ensure balance on those variables. The restricted randomization technique of Hayes and Moulton [46] will be implemented within the four strata to assure overall school balance across the three experimental groups. Each of the 42 schools will be randomly assigned to one of the three study conditions such that all selected adolescent girls from a particular school will receive the same intervention (to reduce contamination). In sum, of the 42 secondary schools, 14 will be randomly assigned to receive a YDA (*n* = 420 students), 14 to combination intervention: MFG + YDA (n = 420 students), and the final 14 to the usual care intervention comprising of standard health and sex education provided in secondary schools (*n* = 420 students) (Fig. 2).

Power analyses were generated using the two-group repeated measures modules in NCSS PASS 14 to compute minimum detectable effect sizes for the primary analysis. The study will begin with 1260 participants from 42 schools evenly assigned to the three study groups. Conservatively assuming 20% attrition, data from 1008 participants will be available for analysis at all time points. Given anticipated within-school correlations, the effective sample size (ESS) was lowered to be ESS = N/DEFF, where DEFF is the design effect or variance inflation attributable to using correlated data. Conservatively assuming DEFF = 4.00, ESS = 1008/4 = 252, division of 252 by 3 study groups yielded 84 per study arm. Assuming α = .017, power = .80, and ESS = 168, we computed for binary STI and risk outcome variables used to evaluate the minimum detectable (inverted) odds ratio (*OR*), proportion difference (*pdiff*), and standardized proportion difference (*h*) for the proposed time-averaged 2-group paired comparisons for outcome base rates ranging from 10 to 50%. For continuous mental health outcomes, we computed the standardized mean difference *d*. Since within-subject correlations among the various outcome measurements following intervention are unknown, the within-subject correlation ρ was varied between .20 and .70. Effect size estimates for our primary analyses range from .33 to .45, which are between benchmark thresholds of .20 and .50 for small and medium standardized effect sizes [47], suggesting that our proposed primary analyses have sufficient power to detect small to medium effects across a variety of analysis conditions.

### Ethics and consent

Written informed assent and consent will be obtained from the adolescent participant and their caregiver, respectively. The processes for adult caregivers and adolescents will be separate to avoid any coercion. Consent and assent forms will be translated and back-translated between English and Luganda. While adolescent participants will be English speaking, some may be more comfortable using Luganda. Therefore, the assent process will be conducted in English or Luganda depending on participants’ English proficiency. Interviewers will make it explicitly known to the participants that they may refuse to answer a question or decline to undergo a procedure, at any time. The study team will receive training on Good Clinical Practice (GCP) so that sensitive topics and issues are handled appropriately. Additionally, for questions measuring sensitive behaviors like sexual risk-taking and mental health, we will use computer-assisted self-interviews where the participant takes the survey herself on a mini laptop/ipad, providing additional privacy and confidentiality.

In line with the Uganda HIV disclosure policy, study participants will receive their HIV results independent of their caregiver. Upon receipt of the child’s consent to do so, a parent or guardian will then be informed. Research Assistants will contact girls who test positive 30 days after telling them of their HIV status, to confirm that they have connected with follow up care and have access to comprehensive care services. Girls testing positive for HIV, STIs (Gonorrhea, Trichomonas, or Chlamydia) or pregnancy will be referred for care and support. All study procedures were approved by the Washington University in St. Louis Review Board (IRB) – home institution of the PI – and by in-country local IRBs in Uganda: Uganda Virus Research Institute (UVRI), and Uganda National Council of Science and Technology (UNCST).

### Quantitative assessment and analytic plan

Assessments will occur at baseline, 12-, 24-, and 36-months (Fig. 2) and will take place at the participant’s home or at school (in a private room) with each lasting about 60 min. Interviews will be conducted in English or Luganda depending on participants’ English proficiency. All interviewers will be fluent in both languages. A list of standardized instruments that will be included in the main statistical analyses are outlined in Table 1. All measures used have been or will be pre-tested and made culturally appropriate to the Ugandan context. For questions measuring sensitive behaviors, computer assisted self-interviews will be implemented. Non-sensitive questions will be interviewer-administered using Qualtrics.

**Table 1:** Suubi4Her Instruments

Variable	Measurement	Reliability	Time point
Demographics			
Age; orphan status (single vs. double); socioeconomic status; family composition/structure; caregiver educational level	Socio-demographic questionnaire	n/a	B, 12, 24, 36
Moderators			
Rural/urban/semi-urban; exposure to outside HIV/STI-related programs; economic/household income; asset accumulation			B, 12, 24, 36
Primary Outcomes			
HIV/Sexual risk taking behavior	Time-line Follow Back (TFLB) [49]	n/a	B, 12, 24, 36
Mental Health Functioning	Beck Hopelessness Scale [50]	0.79	B, 12, 24, 36
	Adapted Tennessee Self-Concept Scale [51]	0.81	
	Adapted Child Depression Inventory [52]	0.65	
Secondary Exploratory Outcomes & Potential Mechanisms of Change			
STI	Biomedical data: Gonorrhea, Chlamydia, Trichomonas,	n/a	B, 12, 24, 36
HIV, Viral load^a^, CD4 count^a^, Pregnancy Incidence	Biomedical data: HIV, Viral load^a^, CD4 count^a^, Pregnancy test	n/a	B, 12, 24, 36
Self-efficacy	Adapted Tennessee Self-Concept Scale (TSC-2) [51]	0.81	B, 12, 24, 36
Education plans/aspirations	Adapted Monitoring the Future Survey [53]	n/a	B, 12, 24, 36
Motivation to participate	Questions tested in previous studies [20, 23–25]	n/a	B, 12, 24, 36
Family Support	Social Support Behaviors Scale (SS-B) [54]	0.77	B, 12, 24, 36
	Family Cohesions Scale [55], Krauss Interview [56] Parent Child Relationship Inventory (PCRI) [57]	0.69	
		0.91	
Family Stability	Socio-demographic questionnaire	n/a	B, 12, 24, 36
Self-esteem	Rosenberg Self-Esteem Scale [58]	0.77–0.88	B, 12, 24, 36
Attitudes towards gender roles; Decision-making; Communication	Gender Norm Attitudes Scale [59]	0.67–0.70	B, 12, 24, 36
HIV/STI knowledge	HIV/STI knowledge	0.80	B, 12, 24, 36
Condom Negotiation Self-Efficacy	Condom negotiation self-efficacy scale [60]	0.80	B, 12, 24, 36
Sexual Communication Skills	Sexual Communication Scale [59]	0.80	B, 12, 24, 36
Social Support	MSPSS [61]	0.84	B, 12, 24, 36
Savings Deposits	Bank statements	n/a	B, 12, 24, 36
Financial Literacy	Financial Literacy knowledge [62]	0.80	B, 12, 24, 36
Access to services	RBA Services [63]	.66–.83	B, 12, 24, 36
Cost of staff time, supplies, overhead for YDA and for MFG	Project records; Admin. review	n/a	ongoing

^a^For participants testing positive for HIV

The two primary outcome measures for the study are: 1) change in HIV risk behavior (occurrence of risky sexual behaviors as measured by Timeline Follow Back (TLFB) for Specific Aim 1; and 2) change in mental health functioning (continuous variables of depression, self esteem, and hopelessness as measured by Beck’s Hopelessness Scale and adapted versions of the Child Depression Inventory and Tennessee Self-Concept Scales, all of which have been pre-tested in earlier Suubi trials, demonstrating strong internal consistency [20, 23–25, 39]) (Table 1) for Specific Aim 2. Secondary exploratory outcomes include HIV and STI incidence (including Gonorrhea, Trichomonas, and Chlamydia), pregnancy incidence, educational attainment, financial savings behavior, gender attitudes, and self-esteem. All study tools were translated into Luganda and back-translated into English.

### Primary analyses

An intent to treat (ITT) approach will be employed. Prior to initial analyses, the study team will determine collectively whether to estimate marginal population average effects versus conditional subject-specific effects. Interest in population average vs. subject-specific effects and the relative importance of explicitly estimating school-level and person-level variance components will be considered during these discussions.

For marginal effects estimation, generalized estimating equations (GEE) will be used to perform the proposed primary analyses. The alternating logistic regression (ALR) approach implemented in SAS PROC GENMOD will be employed to address the 3-level clustering of observations within participants and participants within schools. Multiple imputation (MI) will be used prior to fitting GEEs to include cases with partial data under the missing-at-random (MAR) assumption. The QIC statistic will be used to select an optimal working correlation structure for GEEs.

For conditional effects estimation, multilevel or similar (e.g., latent growth curve) models will be used. Initial three-level models containing random intercepts for schools and random intercepts and slopes for girls will be considered with cases with partial outcome data automatically included under the missing-at-random (MAR) assumption. Alternative covariance structures may be considered to facilitate convergence and improve model fit. Covariance structures will be compared using information-theoretic criteria such as the BIC to select the optimal covariance structure.

HIV risk behavior is the primary outcome to address Specific Aim 1. Mental Health functioning is the primary outcome to address Specific Aim 2. We hypothesize that following baseline: 1) YDA will have lower odds of HIV risk behavior and higher mean mental health functioning vs. control participants; 2) YDA + MFG will have lower odds of HIV risk behavior and higher mean mental health functioning vs. control participants, and 3) YDA + MFG will have lower odds of HIV risk behavior and higher mean mental high functioning vs. YDA participants. For each primary outcome, these hypotheses will be tested by three pairwise planned time-averaged comparisons of post-baseline measurements. Alpha (α) will be set at .05/3 = .017 for these three planned comparisons to maintain a nominal α = .05. Any additional post-hoc comparisons (e.g., paired comparisons of groups at each time point) will maintain nominal alpha of .05 through the use of multiple comparison adjustment methods (e.g., simulation-based stepdown methods).

### Secondary exploratory analyses

We will also describe the proportion of new HIV cases among girls who are HIV-negative at study entry and who seroconvert during the study. Although our study is not formally powered to test for differences in the proportion of HIV incident cases across the three study groups, we will explore the effect of YDA and YDA + MFG on the proportion of HIV incident cases observed across the post-baseline study period. The same multilevel modeling and GEE approaches described above will be used to compare the odds and mean levels of other secondary outcomes across study groups for binary and continuous outcomes, respectively. To explore hypothesized mechanisms of change, secondary exploratory analyses will also investigate whether mental health constructs at 12 and 24 months mediate the relationship between intervention group assignment and HIV risk outcomes at 24 and 36 months and whether HIV-serostatus moderates these associations. Exploratory analyses will also examine the potential mediating effects of other variables such as family support, self-efficacy, and condom negotiation skills on primary outcome measures.

### Cost analysis

Following standard practice of measuring cost-effectiveness of interventions, we will measure costs on a per person basis. The intervention costs will include all program costs, including the YDA savings match as well as all costs incurred for running the program. Research costs will not be included. Costs from multiple years will be adjusted for inflation, depreciation, and discounting. The outcomes analyses described above will be used to estimate how much Combined Intervention (YDA + MFG) vs YDA-alone increased particular outcomes, such as schooling. The per-person costs of YDA + MFG, and YDA alone will then be divided by the relevant effect sizes to produce estimates of cost-effectiveness. For outcomes that are measured similarly in other intervention studies, such as improvements in education or health, we will be able to compare the cost-effectiveness of YDA-alone and Combination intervention (YDA + MFG) to other interventions in developing country settings.

## Discussion

There is an increasing interest in and use of social safety nets to achieve health outcomes for children. At the same time, there is need to understand how social interventions can complement these economic programs for enhanced impact. The present study protocol describes a three-arm cluster randomized-controlled trial with the primary outcomes of analysis as HIV risk and mental health functioning among school-going adolescent girls. The study will also provide critical information on cost, comparing budget expenditures and adolescent outcomes across study arms, indicating which arm provides better value for money. The findings will yield important insights on the effectiveness of asset-promotion programs and MFG dialogues to reduce HIV risk and improve behavioral health while also exploring the ways in which mental health may serve as a mediator to the success of HIV risk reduction programs for adolescent girls in Uganda.

We do not anticipate any major threats to study implementation though we recognize potential concerns and have adapted accordingly. We have a stringent retention plan for attendance at the MFG and Financial Management sessions. We expect to achieve enrollment and retention goals (90% at 36-month post intervention, a statistic based on our current and previous studies among school going children in the same study area – see *Study Population, Recruitment, and Retention*). However, we are aware that older adolescents are more likely to leave school and migrate for work. As such, we have put in place detailed tracking procedures including each participant providing names, physical addresses, and phone numbers for a minimum of three relatives or friends who would always know the whereabouts of the participant. Moreover, should our recruitment, enrollment, or retention rates deviate from anticipated goals, the study team, comprising both the research and field implementation teams, will determine collectively how best to adjust the field outreach activities.

Another limitation of the proposed study is that the YDA intervention may only appeal to families that feel they have expendable resources to contribute to savings accounts. Others may see YDAs, which defer consumption, to be impractical and detrimental to meeting their basic needs. However, with the proposed 12 sessions of financial management tailored specifically to the needs of adolescent girls and their families in Uganda, we expect that even those with the fewest resources will be equipped to make well-informed saving and investment decisions—however modest the amount. We will carefully track the saving behavior, including deposit frequency and withdrawals of each family enrolled in the study.

Despite these limitations, Suubi4Her, as a combination intervention, is innovative and poised to make a contribution to the literature on HIV prevention for adolescent girls in SSA for a number of reasons. First, it applies a theoretically guided economic empowerment intervention that uses incentivized/matched savings accounts. These accounts have been widely used with a younger primary-school going population and demonstrated positive effects but have never been used with older adolescents nor explicitly combined with an evidence-based approach to enhancing youth behavioral health. Increasing the economic resources of adolescent girls, while emphasizing the importance of girls’ education through a family strengthening approach, may help lower HIV risks, improve mental health functioning, and increase opportunities for adolescent girls. Moreover, the MFG method is culturally consistent with the sub-Saharan (and Ugandan) collective approach of supporting and raising children. Second, given that sexual behavior and mental health are often stigmatized and prone to misreport, exclusive reliance on self-report can lead to misclassification, masked intervention effects, and ambiguity when study findings are interpreted [48]. Suubi4Her addresses this concern by measuring sexual risk-taking behaviors objectively using the following biomedical data: new HIV infection, STIs (including Gonorrhea, Trichomonas and Chlamydia) and pregnancy tests. The study will also address this issue by using computer assisted self-interview to obtain more reliable responses on sensitive topics, such as sexual risk taking behaviors and mental health. Lastly, Suubi4Her builds upon a decade of partnerships (including but not limited to schools, financial institutions, government departments, clinics, and community organizations) to ensure interventions are rooted in a practical and localized understanding of the needs of older adolescent girls. These partnerships help ground the study in the community, building the capacity and helping to ensure eventual scale up, if findings warrant.

## Conclusion

Suubi4Her is one of the first prospective studies to examine the impact and cost of a combination intervention integrating economic and social components to reduce known HIV risk factors and improve mental health functioning among adolescent girls, while exploring mental health as a mediator in HIV risk reduction. The findings will illuminate the pathways that connect economic need, mental health, family support, and HIV risk. If successful, the results will promote holistic HIV prevention strategies to reduce risk among adolescent girls in Uganda and potentially the broader SSA region.

### Trial status

At the time of manuscript submission, the trial had received IRB approvals and was making preparations for recruitment. The study is ongoing.
